# Optical analysis of the behavior of sealants under mechanical, thermal and chemical stress

**DOI:** 10.1038/s41598-021-87288-7

**Published:** 2021-04-07

**Authors:** Christina Erbe, Florian Deckers, Irene Schmidtmann, Julia Heider, Heinrich Wehrbein

**Affiliations:** 1 Department of Orthodontics, University Medical Centre of the Johannes Gutenberg-University, Augustusplatz 2, 55131 Mainz, Germany; 2 Institute for Medical Biostatistics, Epidemiology and Informatics (IMBEI), University Medical Centre of the Johannes Gutenberg-University, Mainz, Germany; 3 Department of Oral and Maxillo-Facial Surgery, University Medical Center of the Johannes Gutenberg-University, Mainz, Germany

**Keywords:** Oral diseases, Dental public health, Preventive dentistry, Dentistry, Orthodontics, Fixed appliances

## Abstract

Regarding their resistance five sealants were tested in vitro after experiencing mechanical, thermal and chemical stress. Included for testing were two fluoride varnishes: Fluor Protector [FP] (Ivoclar Vivadent) and Protecto CaF2 Nano One-Step Seal [PN] (BonaDent) and three fluoride-composite filled sealants (with acid etch technique): Clinpro XT Varnish [CP] (3 M Espe), Pro Seal [PS] & Light Bond [LB] (Reliance Orthodontic Products) and a positive control group [CG] Tetric EvoFlow (Ivoclar Vivadent). The sealants were applied on 180 bovine teeth (n = 10/ sealer) in a standardized manner after bracket bonding. Mechanical pressure and its effect by simulating different time points and standardized electric cleaning protocol was tested first. Followed by thermal burden due to varying thermal stress and thirdly change in pH stress imitating chemical exposure were examined separately. A digital microscope and a grid incisal and apical to the brackets (n = 32 fields) was used to standardize the optical analysis. Material loss due to mechanical stress compared to CG (score 0.00) was CP (1.2%), FP (21.5%), LB (22.2%) and PN (81.1%). No significant difference to CG presented PS. Material loss due to thermal stress was CP (0.5%), PS (2%), FP (2.6%), LB (3.1%) and PN (39.9%). Material loss due to chemical stress was FP (1.8%), PS (2.1%), LB (5.5%) and PN (39.6%). No significant difference to CG presented CP. Only PS and CP had optically provable, good resiliance to mechanical, thermal and chemical stress. Significantly poorer outcomes in particular showed PN.

## Introduction

Fixed appliances for the removal of malpositioned teeth are used in orthodontics for both adolescents and adults. Even today, difficult oral hygiene and the associated increased accumulation of plaque and food residues during therapy with multibracket appliances (MBA) represent an additional caries risk^1^. The development of demineralization, causing white, opaque changes in the enamel are known as white spot lesions (WSL), during treatment with MBA is a frequent and undesirable side effect and can occur after just 4 weeks^2, 3^.

In recent years, increased attention has been paid to sealing of the buccal surfaces and the use of special sealants and fluoride varnishes. These products are expected to provide long-term caries prevention and additional protection against external stresses. The various manufacturers promise protection between 6 and 12 months after a single application. In the current literature different results and recommendations can be found regarding the preventive effect and benefit for the application of such products. In addition, there are various statements regarding their resistance to stress. Five frequently used products were included: the composite based sealants Pro Seal, Light Bond (both Reliance Orthodontic Products, Itasca, Illinois, USA) and Clinpro XT Varnish (3 M Espe AG Dental Products, Seefeld, Germany). Also investigated were the two fluoride varnishes Fluor Protector (Ivoclar Vivadent GmbH, Ellwangen, Germany) and Protecto CaF2 Nano One-Step-Seal (BonaDent GmbH, Frankfurt/Main, Germany). A flowable, light-curing, radiopaque nanohybrid composite was used as the positive control group (Tetric EvoFlow, Ivoclar Vivadent, Ellwangen, Germany).

These five frequently used sealants were investigated in vitro towards their resistance after experiencing mechanical pressure, thermal burden and chemical exposure causing demineralization and consequently WSL.

The following hypotheses will be tested:

- Null hypothesis: Mechanical, thermal and chemical stresses do not affect the sealants investigated.
- Alternative hypothesis: Mechanical, thermal and chemical stresses affect the sealants investigated.

## Material and method

192 bovine front teeth were used in this in vitro study. The bovine teeth were extracted from slaughter animals (slaughterhouse, Alzey, Germany). The selection criteria for bovine teeth were caries- and defect free, vestibular enamel without discoloration of the tooth surface and sufficient size of the tooth crown^4^. Storage was in a 0.5% chloramine B solution^5, 6^. Before and after bracket application, the vestibular smooth surfaces of all bovine teeth were additionally cleaned with an oil- and fluoride-free polishing paste (Zircate Prophy Paste, Dentsply DeTrey GmbH, Konstanz, Germany), rinsed off with water and dried with air^5^. Metal brackets made of nickel-free stainless steel were used for the study (Mini-Sprint Brackets, Forestadent, Pforzheim, Germany). All brackets used UnitekEtching Gel, Transbond XT Light Cure Adhesive Primer and Transbond XT Light Cure Orthodontic Adhesive (all 3 M Unitek GmbH, Seefeld, Germany). After bracket application, the vestibular smooth surfaces were cleaned again with Zircate Prophy Paste to remove any adhesive residue^5^. To simulate the ideal clinical situation during mechanical cleaning, a 2 cm long single archwire piece (Forestalloy blue, Forestadent, Pforzheim, Germany) was applied to the bracket with a preformed wire ligature (0.25 mm, Forestadent, Pforzheim, Germany).

A total of five sealants were investigated in this study. In selecting the materials, reference was made to a current survey. In Germany, 985 dentists were asked about the sealants used in their orthodontic practices. The most mentioned five out of the eleven materials were selected. All materials were used strictly according to the manufacturer's instructions. Tetric EvoFlow served as the positive control group.

Based on a self-developed time module to simulate the average mechanical load, all sealants were subjected to a mechanical load and subsequently tested. An electrical toothbrush, Oral-B Professional Care 1000 (Procter & Gamble GmbH, Schwalbach am Taunus, Germany), was used in this study to simulate the mechanical load. A visual pressure check illuminates when the physiological contact pressure (2 N) is exceeded. Oral-B Precision Clean EB 20 (Procter & Gamble GmbH, Schwalbach am Taunus, Germany) were used as toothbrush heads. The brush head was renewed for each test group (i.e.6 times). During the study, the same toothpaste (Elmex, GABA GmbH, Lörrach, Germany) was always used in order to minimize its influence on the results^7^. In a preliminary experiment, the average pea-sized amount of toothpaste was measured and calculated using a microbalance (Pioneer analytical balance, OHAUS, Nänikon, Switzerland) (385 mg). The brush head was moistened with distilled water, moistened with 385 mg average toothpaste and passively positioned on the vestibular tooth surface. The mechanical load was applied with constant pressure and reciprocal forward and backward movements of the brush head. The exposure time was checked to the second. The electric toothbrush was always guided by the same examiner in all test series. The visual pressure control was used to ensure that the physiological contact pressure (2 N) was not exceeded. After 30 min of use, the toothbrush was fully recharged to ensure consistent and full performance. After brushing, the teeth were cleaned for 20 s with a mild spray of water and then dried with air^8^.

The time module used is based on the assumption that the average cleaning time is 2 min^9, 10^. This corresponds to a cleaning time of 30 s per quadrant. For an average dentition, a full dentition of 28 teeth, i.e. 7 teeth per quadrant, is assumed. Per tooth there are 3 relevant tooth surfaces for the toothbrush: buccal, occlusal and oral. The mesial and distal approximal tooth surfaces should be cleaned with dental floss or similar but are usually not accessible for the toothbrush and can therefore be neglected here. With a cleaning time per quadrant of 30 s, an average cleaning time of 4.29 s per tooth can be assumed. This corresponds to a time of 1.43 s per tooth surface. In summary, it can be assumed that the average cleaning time of a tooth surface per cleaning procedure is approx. 1.5 s. If one considers the vestibular tooth surface treated with a smooth surface sealant, a daily cleaning load of 3 s on average can be assumed for twice daily tooth cleaning. This would correspond to 21 s per week, 84 s per month, 504 s every six months and can be continued as desired. In this study the cleaning exposure after 1 day, 1 week, 6 weeks, 3 months and 6 months was simulated and investigated.

In order to simulate the temperature differences occurring in the oral cavity and the associated stresses, artificial ageing was simulated with a thermal cycler. In this study the thermal cycling load (Circulator DC10, Thermo Haake, Karlsruhe, Germany) between 5 °C and 55 °C at 5000 cycles and an immersion and dripping time of 30 s each was carried out simulating the exposure and ageing of the sealers for half a year^11^. The thermal baths were filled with distilled water. After reaching the initial temperature, all tooth samples oscillated 5000 times between the cold pool and the heat pool. The immersion time was 30 s each, followed by a 30 s drip and transfer time.

In order to simulate the daily acid attacks and mineralization processes on the sealants in the oral cavity, a pH change exposure was carried out. The solutions selected were the Buskes^12, 13^ solution described many times in the literature. The pH value of the demineralization solution is 5 and that of the remineralization solution is 7. The components of the remineralization solutions are calcium dichloride-2-hydrate (CaCl2-2H2O), potassium dihydrogen phosphate (KH2PO4), HE-PES (1 M), potassium hydroxide (1 M) and aqua destillata. The components of the demineralization solution are calcium dichloride -2-hydrate (CaCl2-2H2O), potassium dihydrogen phosphate (KH2PO4), methylenediphosphoric acid (MHDP), potassium hydroxide (10 M) and aqua destillata. A 7-day pH-cycling was carried out^5, 14^. All groups were subjected to 22-h remineralization and 2-h demineralization per day (alternating from 11 h-1 h-11 h-1 h), based on pH cycling protocols already used in the literature^15, 16^. Two large glass bowls (20 × 20 × 8 cm, 1500 ml^3^, Simax, Bohemia Cristal, Selb, Germany) with lids were chosen as containers in which all samples were stored together. The covers were only removed when the samples were changed into the other tray. The samples were stored at room temperature (20 °C ± 1 °C) at a constant pH value in the glass dishes^5, 8, 17^. The pH value of the solution was checked daily with a pH meter (3510 pH Meter, Jenway, Bibby Scientific Ltd, Essex, UK). Every second day, the complete solution was renewed, which prevented a possible drop in the pH value. When changing samples from one dish to the other, the samples were carefully cleaned with distilled water and then dried with an air jet to avoid mixing the solutions. After the 7-day pH cycling, the samples were stored in the hydrophorus and evaluated directly under the microscope. For optical analysis in this study the digital microscope VHX-1000 with VHX-1100 camera, the movable tripod S50 with VHZ-100 optics, the measuring software VHX-H3M and the high-resolution 17-inch LCD monitor (Keyence GmbH, Neu-Isenburg, Germany) were used. Two examination fields with 16 individual fields each could be defined for each tooth, once incisal and apical of the bracket base. As a result, a total of 32 fields per tooth and 320 fields per material were defined in a test series. To best address the everyday important clinical relevance and approach to visual assessment of sealants with the naked eye, each individual field was viewed under the digital microscope with a 1000 × magnification, visually evaluated and assigned to an examination variable. The examination variables were 0: material = the examined field is completely covered with sealing material, 1: defective sealant = the examined field shows a complete loss of material or a considerable reduction at one point, where the tooth surface becomes visible, but with a remaining layer of the sealant, 2: Material loss = the examined field shows a complete material loss, the tooth surface is exposed or *: cannot be evaluated = the examined field cannot be represented optically sufficiently or the sealer is not sufficiently applied, then this field fails for the test series.

### Statistics

The statistical analysis was performed with SAS (9.3 2002–10 Cary, NC, USA) with a generalized estimation equation according to a Poisson regression model (post-hoc: Tukey Kramer test).

A total of 32 fields (16 incisal and 16 apical of the bracket) were checked per tooth. In each field, the material was evaluated as complete (0 points), defective / insufficient (1 point) or material loss (2 points). A score was determined from all 32 fields of each tooth at each measurement time by summing the point values. This score could assume values between 0 (all fields completely covered) and 64 (all fields with complete loss of material), thus two defective / insufficient fields were evaluated as one field with loss of material. For individual fields in the three examinations, evaluation was not possible for technical reasons. Therefore, a relative score was determined: Yijk be the score for the k-th tooth, at time j, using material and Nijk the number of evaluable fields, the relative score is defined ij = Yijk/2*Nijk and can be understood as the proportion of material loss. A Poisson distribution was assumed for the distribution of the score. A Poisson regression model was adapted using generalized estimation equations to describe the dependence of damage on material and duration of mechanical stress. The use of generalized estimation equations takes into account the fact that there were multiple measurements for each tooth. The global significance level was set at 5% (α ≤ 0.05) per test. If significant material effects or time effects were observed, post-hoc comparisons with Tukey–Kramer adjustment were performed.

## Results

### Mechanical stress

**Figure 1:**
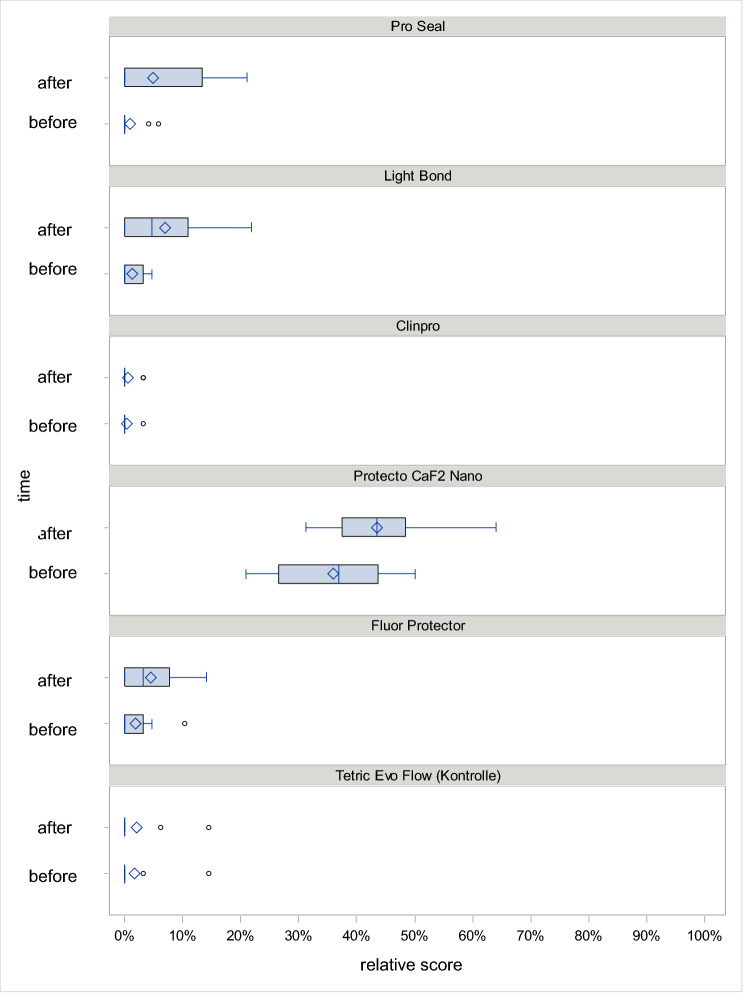
Boxplot diagram for the distribution of the relative material loss score over the different points in time, normalized to the number of evaluable surfaces under mechanical load.

Only four materials were included in the mechanical load modelling, since no material loss was observed on any surface or at any time with Tetric EvoFlow and the material loss was so slight with Pro Seal that too many cells contained the score value = 0. Averaged over all times, the expected proportion of material loss under mechanical stress was 1.2% for Clinpro XT Varnish, 21.5% for Fluor Protector, 22.2% for Light Bond and 81.1% for Protector CaF2 Nano (*p* < 0.0001) (Fig. 1).

Clinpro XT Varnish and Protecto CaF2 Nano showed a significant difference (*p* < 0.0001) between all materials under mechanical stress and all other sealants. Light Bond showed a significant difference to Clinpro XT Varnish and Protecto CaF2 Nano (*p* < 0.0001), but no significant difference to Fluor Protector. Fluor Protector itself showed a significant difference to Clinpro XT Varnish and Protecto CaF2 Nano (*p* < 0.0001).

### Thermal stress

**Figure 2:**
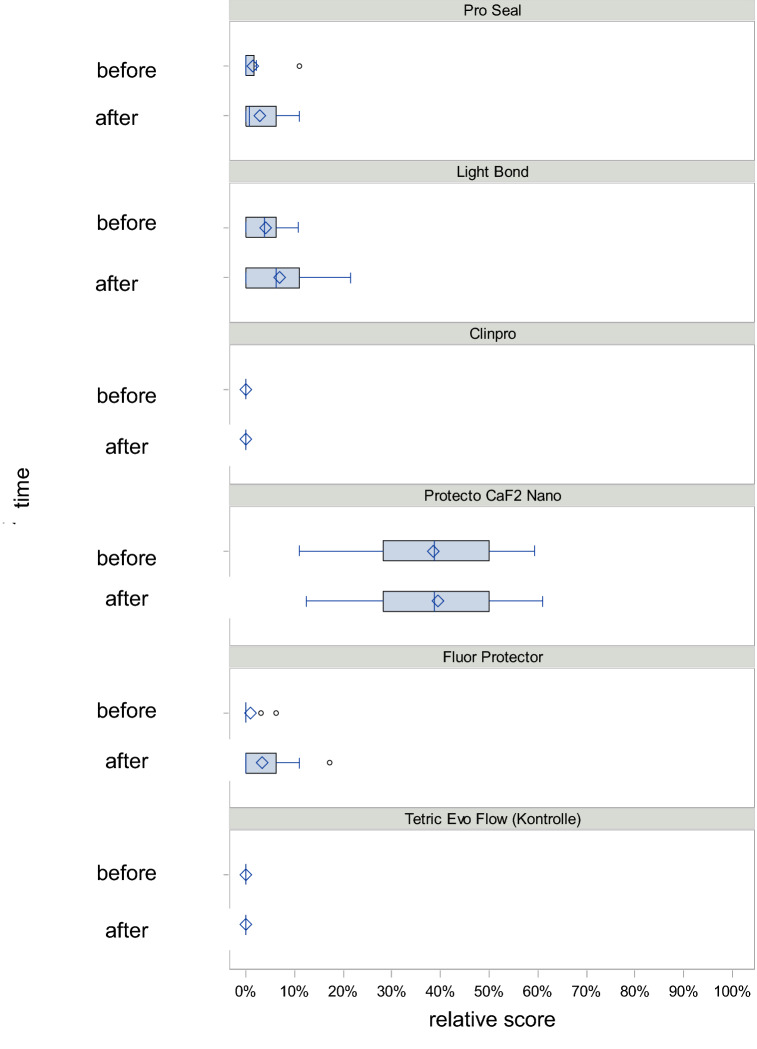
Boxplot diagram for the distribution of the relative score before and after the test under thermal stress.

Only five materials were included in the thermal loading, since no significant material loss was observed with Tetric EvoFlow. Averaged over all times, the expected proportion of material loss during thermal loading was 0.5% for Clinpro XT Varnish, 2% for Pro Seal, 2.6% for Fluor Protector, 3.1% for Light Bond and 39.9% for Protector CaF2 Nano (*p* < 0.0001) (Fig. 2).

When comparing all materials with each other under thermal stress, Protecto CaF2 Nano showed a significant difference to all other materials (*p* < 0.0001). Clinpro XT Varnish, Pro Seal, Fluor Protector and Light Bond showed no significant difference among each other.

### Chemical stress

**Figure 3:**
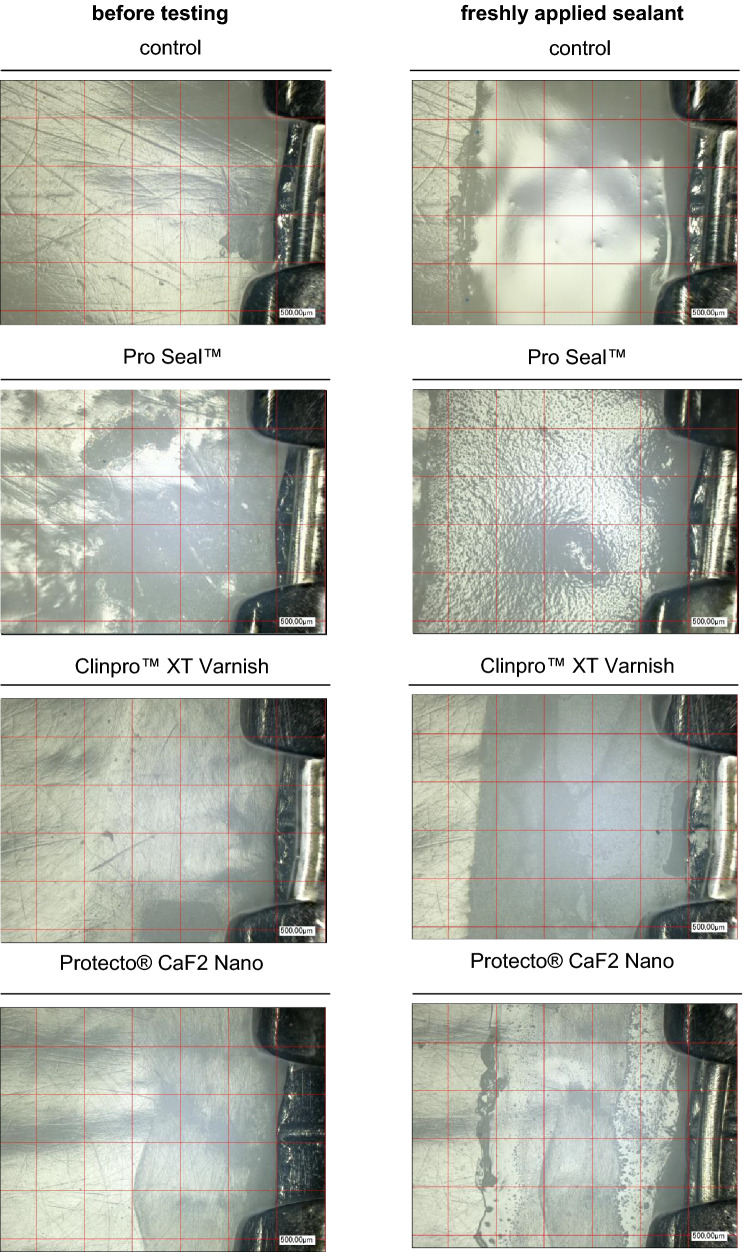

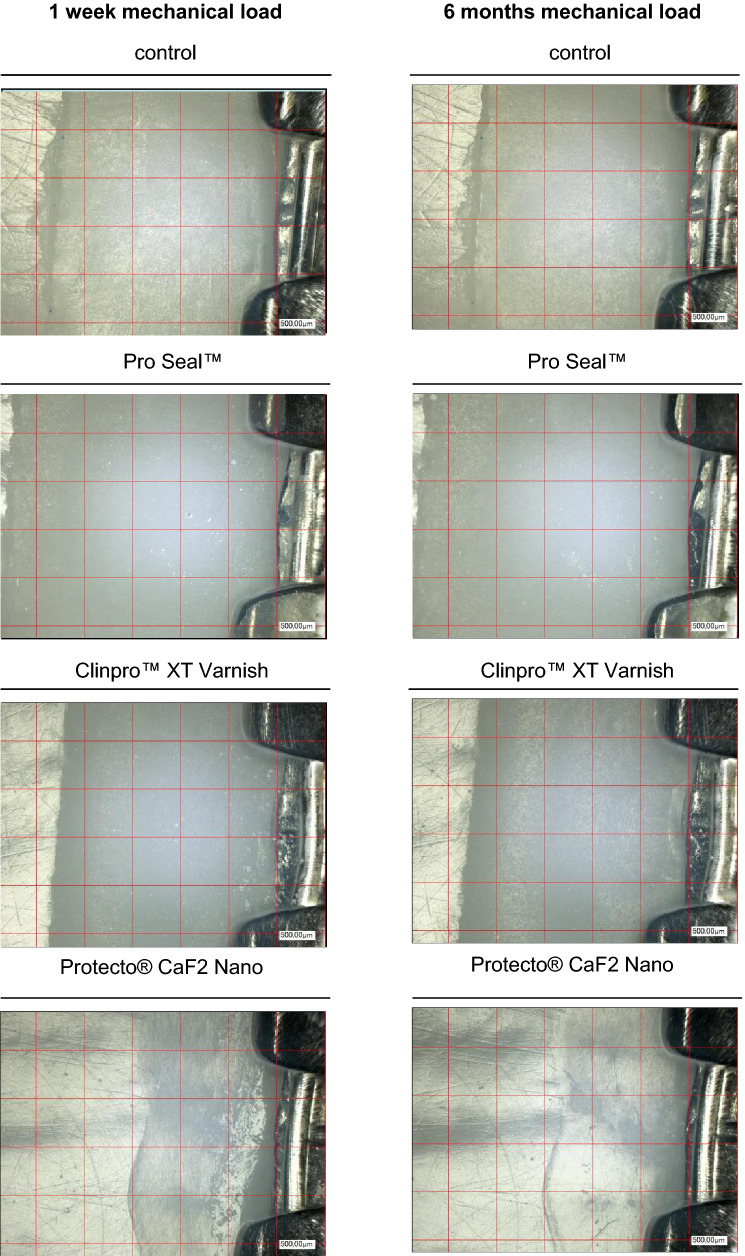
Boxplot diagram for the distribution of the relative score before and after the test under chemical exposure.

Only four materials were included in the modelling during the chemical exposure, as no loss of material was observed on any surface and at any time with Tetric EvoFlow. Also with Clinpro XT Varnish, the material loss was so small that too many cells contained the score value = 0. Averaged over all time points, the expected proportion of material loss during chemical exposure was 1.8% for Fluor Protector, 2.1% for Pro Seal, 5.5% for Light Bond and 39.6% for Protector CaF2 Nano (*p* < 0.0001) (Fig. 3).

When comparing all materials with each other under chemical exposure, Protecto CaF2 Nano showed a significant difference to all other materials (*p* < 0.0001). Among each other Pro Seal, Fluor Protector and Light Bond showed no significant difference.

## Discussion

In numerous product studies bovine teeth are used instead of human teeth. In a 2015 study by Teruel et al. concluded that bovine teeth are well suited to the examination of dental products instead of human teeth^18^. In this study, bovine teeth were used to obtain a sufficient number of teeth with caries and defect free enamel without discoloration of the tooth surface. In comparison to human teeth, possible influences such as previous treatments, fluoridation measures or other changes to the tooth surface can also be neglected.

The literature contains numerous studies in which mechanical loads caused by brushing are performed and investigated^17, 19–25^. Various studies describe mechanical loading with a brushing simulator or manual brushing by test persons or patients. When using a brushing simulator, a certain number of samples are usually clamped in a device and automatically cleaned after setting the cleaning movement, contact pressure and cleaning time. However, studies using a simulator for brushing vary in their protocols^17, 20, 21^. The advantage of a cleaning machine is the guarantee of a uniform cleaning movement and a controlled load due to the contact pressure. Behnan et al., on the other hand, did not use a cleaning simulator in 2010 and described a simple cleaning of the specimen with an electric toothbrush at intervals of 2 s each with subsequent rinsing of the samples under tap water^22^. By manually guiding the toothbrush, individual brushing factors could be taken into account in terms of brushing technique and contact pressure compared to an in-vitro brushing appliance. This would reflect the conditions and practical aspects more realistically than an brushing simulator. In the present study, the electric toothbrush was always manually guided by only one examiner in all test series, following the procedure of Behnan et al. 2010. This made it possible to take into account an individual factor of manual brushing in terms of brushing technique and contact pressure. Slightly different loads due to brushing movement or contact pressure when manually guiding the toothbrush cannot be excluded with this study model.

The average time for oral hygiene and the temporal simulation of a certain mechanical load in in vitro studies is a discussed topic in the literature. An average cleaning time between 46 and 83.5 s is described^26, 27^. In cleaning studies, cleaning times of 60 or 120 s are often chosen^7, 23, 28^. Most of them used a cleaning time of 2 min^9, 10, 14, 28^. Based on these recommendations, this study also assumed an average cleaning time of 2 min. In order to simulate the temperature differences occurring in the oral cavity and the associated stresses, artificial ageing of products is simulated in the literature with thermocycling. Dental products should resist thermal stress caused by temperature changes in the mouth. Water storage and thermal cycling are a method frequently used and described in the literature for in vitro investigations with which products can be examined experimentally with regard to their adhesive strength under comparable conditions in the oral cavity^14, 29^.

All products in this study should be able to withstand thermal stress and temperature changes, especially those caused by respiration^30^ and the daily intake of food^31^. In 1999, Gale et al. compared 130 thermocycling studies with their parameters in a review paper. The total temperature range was between 0 and 100 °C, the average value was between 5 and 55 °C. The temperature range was between 0 and 100 °C, and the average value was between 5 and 55 °C. They also recommended a number of 10,000 cycles for the load simulation of one year^11^. According to this recommendation, 5000 cycles simulate the load of half a year. Momoi et al. described in 1990 the effect of thermal loading on composites with the formation of marginal gaps and leaks^32^. The products used in this study also contain polymers. Therefore, material tension and chipping of parts of the sealant is possible^14^. The draining time or retention time per water bath is given in the literature as approx. 30 s on average^14^. The study model of thermal stress used in this study can therefore be regarded as a standard procedure based on the current literature.

In order to simulate the daily acid attacks and mineralization processes on dental products in the oral cavity, the literature recommends an alternating pH load. Dental products should resist chemical exposure caused by daily acid attacks in the mouth. Demineralization and remineralization solutions are a procedure frequently used and described in the literature. Dental products can thus be examined under comparable conditions in the oral cavity^8, 12, 16, 17, 20^. Different solutions and procedures are described in the literature. In particular, the individual solutions, the pH values, the duration of the procedure and the temperatures of the liquids differ. The method most frequently described in the current literature is based on Buskes et al. or ten Cate et al. lactic acid gelatine systems^33^, hydroxyethylcellulose with lactic acid^34^ or mixtures of diphosphonate and lactic acid^35^. The Buskes demineralization solution is described in the literature as a reliable and frequently used method^36, 37^. The advantage of this method is the continuous formation of enamel lesions with the characteristic zone morphology^12^.

The results of this study confirm the positive properties of Pro Seal described in the literature. This material can be described as a standard product in bracket environment treatment. Similar results were obtained in the in vitro study by Coordes^14^. Various sealants were also tested for their resistance to stress. The sealants Pro Seal and Fluor Protector were tested on n = 120 extracted teeth in 6 groups. After thermal (1000 cycles, 5 °C and 55 °C), mechanical (brushing simulator 1000 cycles) and chemical loading (7 days, ten cate-solution) the samples were cut through and examined light microscopically for the lesion depths. Only the Pro Seal group showed no demineralization with a significant difference of the mean lesion depth compared to the other products. In addition, it was described that all samples were covered with a clearly sealed tooth surface. Fluor Protector, on the other hand, showed demineralization on all tooth samples in the absence of material on the tooth surface^14^. Paschos et al. described a similar situation in an in vitro study. The sealers Pro Seal and Clinpro XT Varnish and the fluoride varnishes Fluor Protector and Protecto CaF2 Nano were also tested on n = 75 extracted molars after a 4-week pH cycling with μCT. All materials showed a protective effect against the formation of enamel lesions compared to an untreated enamel area. However, no lesions occurred only in Pro Seal, the other materials did not completely protect the enamel^38^. Hu and Featherstone also came to similar conclusions and described a caries-protective effect of Pro Seal. In n = 50 extracted wisdom teeth, abrasion stability and demineralization depths were investigated by microhardness measurement under mechanical (15,000 simulated brush strokes) and chemical stress (14 days pH cycling, 6 h demineralization pH = 4.3, 17 h remineralization pH = 7.0)^20, 35^. In 2006, Cain et al. confirmed the caries protection of Pro Seal on n = 40 extracted wisdom teeth after three different demineralization rounds (ten cate-solution), as did Clark^21^ after mechanical and chemical loading on 75 extracted wisdom teeth^21^. The study results from Clinpro XT Varnish partly confirm the positive properties of the sealant described in the literature. In 2014, Silin et al. described the significantly best caries-protective effect of Clinpro XT Varnish compared to other materials^39^ in a randomized blind study with 136 children aged 7–12 years. Clinpro XT Varnish is a relatively new or little studied product compared to Pro Seal. There are only a few studies. Further studies on this subject are desirable. The results of Light Bond in this study are largely comparable to the results of other studies. A caries-protective effect is described in the literature, but with less significance compared to other materials. Thermal, chemical and in particular mechanical stress also led to material losses in this study. Comparable with the results of a study by Paschos et al.^16^, Light Bond also achieved only an average positive effect. 85 extracted human teeth were evaluated microtomographically and microscopically after 30 days of pH cycling (2 h demineralization pH = 4.4, 22 h remineralization neutral solution). There were significant differences within the different groups, with Ortho Conditioner and Fuji Ortho LC exhibiting the lowest lesion values, with an average positive effect of Light Bond^16^. Similar results were also described by Tanna et al. 2009. After mechanical (2 min loading in the toothbrush simulator) and chemical loading (48 h and 72 h pH cycling, ten Cate-solution pH = 4.46), 50% of lesions still occurred in the Light Bond group, whereas 100% occurred in the other groups (Transbond Plus Self Etching Primer (3 M Unitek, Monrovia, CA, USA)) and the control group^8^. The results of the in vivo study of Shinaishin et al.^40^ also showed worse results for Light Bond compared to Pro Seal^40^. However, Light Bond achieved extremely positive results in the studies of Cain et al.^41^, Heinig et al.^42^, Frazier et al.^43^ and Wilson et al.^44^. The results of Fluor Protector in this study are also largely comparable with the results of other studies. Here, too, a caries-protective effect is described in the literature, albeit with a minor significance compared to other materials. Thermal, chemical and especially mechanical stress caused material losses in this study. As already described, the results of the study by Paschos et al. 2014 showed a protective effect of Fluor Protector against the development of enamel lesions, with a lower significance compared to Pro Seal^38^. Buren et al.^17^ also achieved similar results. After mechanical (15,000 simulated cleaning strokes) and chemical exposure (96 h ten Cate-solution, pH = 4.4) the n = 32 extracted molars were examined light microscopically. A significant reduction in lesion depth was observed with Fluor Protector. Pro Seal achieved better results^17^. Seppä et al. compared 1982 Fluor Protector with the effect of Duraphat, with 22600 ppm fluoride content (Colgate Oral Pharmaceuticals, Cologne, Germany). Fluor Protector did not achieve a significantly better success with regard to caries reduction^45^.In contrast, Fluor Protector achieved extremely positive results in the studies of Shafi^46^, Stecksén-Blicks et al.^47^, He et al.^48^ and de Bruyn et al.^49^. Protecto CaF2 Nano One-Step-Seal is a relatively new or little investigated material. There are only isolated studies about this product. The results in this study can be compared with results from previous studies. In the case of thermal, chemical and, in particular, mechanical stress, significant material losses were recorded in the present study. Bechtold et al. also investigated the fluoride varnish Protecto. The protective effect of Protecto and Light Bond was compared in vivo. The enamel surfaces of n = 40 patients with MBA were examined at the beginning and end of treatment with the DIAGNOdent pen (KaVo Dental GmbH, Biberach, Germany) over a period of 6 months. Using this laser fluorescence technology, carious lesions can also be detected at an early stage. Bechtold et al. also did not describe a proven protective effect after a single application over a period of 6 months. Demineralization occurred at the same frequency in all groups^50^. Comparable to the results of the study by Paschos et al.^16^, Protecto achieved the worst results regarding the effectiveness of enamel demineralization prevention. In 2009, Paschos et al. investigated the effectiveness of the enamel demineralisation prevention of Pro Seal, Clinpro Sealant (3 M Espe, St. Paul, MN, USA), Protecto and Fluor Protector in an in-vitro study with n = 90 extracted human molars. After a 7-day pH-cycling histological sections were examined with µCT and QLF. The evaluation showed the following valence order: Pro Seal > Clinpro > Fluor Protector > Protecto. Only Pro Seal showed no lesion or loss of mineralization^16^. Further studies on the material Protecto CaF2 Nano One-Step-Seal are desirable.

The extent to which the different fluoride amounts in the individual materials have an effect on the functional properties with regard to a caries-protective effect may have to be investigated in future studies. The two fluoride varnishes ProtectoCaF2 Nano and Fluor Protector indicate their concentration of soluble fluorides at ~ 0.1%. The contents of the sealants show a fluoride concentration of < 1% sodium fluoride at Light Bond, 5–40% fluoride-containing fillers at Pro Seal and 70–80% plastic-modified glass ionomer cement including fluoroaluminium silicate glass at Clinpro XT Varnish.

On the basis of the results of this study and the expected proportion of material loss of the individual products, the manufacturer's data must be discussed with regard to the duration of protection and the frequency of application. According to Clinpro XT Varnish, the sealer offers "protection for at least 6 months". With regard to the good results after mechanical, thermal and chemical exposure, protection over 6 months seems possible. Pro Seal contains the statement "Protection over the entire treatment period". An exact time indication is not given thus. Regarding the good results in this study a similarly good protection appears as for example Clinpro XT Varnish for 6 months probable. In the manufacturer data of the second product of Reliance Orthodontic Products Light Bond you will find the information "a single application lasts approx. 1 year". With regard to the mediocre results in this study, protection for an entire year appears questionable. In the case of the Fluor Protector fluoride varnish, the manufacturer's instructions contain the following recommendation: "Normally, Fluor Protector is applied every six months by the dentist". With regard to the mediocre results in this study, protection for six months appears questionable. Thermal, chemical and especially mechanical stress caused material losses. The manufacturers of Protecto CaF2 Nano One-Step-Seal promise "protection for approx. 1 year". In view of the poor results in this study, protection for an entire year seems questionable. According to the results of this study, a more closely meshed application frequency than recommended by the manufacturers therefore appears to be sensible for Light Bond, Fluor Protector and especially for Protecto CaF2 Nano One-Step-Seal.

## Conclusion

Only the sealants with acid-etch-technology Clinpro XT Varnish and Pro Seal were optically relevant in all three tests and showed good resistance to mechanical, thermal and chemical influences. In particular, the fluoride varnish Protecto CaF2 Nano One-Step-Seal showed considerably worse test results. According to the results of this study, a more closely meshed application frequency than recommended by the manufacturers appears sensible for Light Bond, Fluor Protector and especially for Protecto CaF2 Nano One-Step-Seal. Certain disadvantages of the materials, such as limited abrasive strength and duration of action, a more cost-intensive treatment and a partly necessary irreversible condensation of the tooth structure, have to be discussed in comparison to the positive caries-protective effect. No statement is made in this study about the functional properties of the materials. This in vitro study, which mimics the clinical situation as closely as possible, proves for the first time the importance of choosing the right sealant, strictly following the manufacturer's instructions and that a single application during multibracket therapy is not sufficient.

## References

[1] Mitchell L (1992). Decalcification during orthodontic treatment with fixed appliances—an overview. Br. J. Orthod..

[2] Lehmann KM, Hellwig E (2005). Zahnärztliche Propädeutik.

[3] O'Reilly MM, Featherstone JD (1987). Demineralization and remineralization around orthodontic appliances: an in vivo study. Am. J. Orthod. Dentofacial. Orthop..

[4] Carli J. Einfluss des Lagerungsmedium auf die Scherfestigkeitswerte mittels “Etch&Rinse”- Technik an humanen und bovinen Schmelz und Dentinfllächen applizierter KOmposite [Dissertation]. Hamburg: Universitätsklinikum Hamburg Eppendorf; 2011.

[5] Rosenbeck KA. Effektivität der Bracketumfeldbehandlung - EIne in vitro Untersuchung [Dissertation]. München: Ludwig-Maximilians-Universität; 2010.

[6] Canbek K, Karbach M, Gottschalk F, Erbe C, Wehrbein H (2013). Evaluation of bovine and human teeth exposed to thermocycling for microleakage under bonded metal brackets. J. Orofac. Orthop..

[7] Saxer UP, Yankell SL (1997). Impact of improved toothbrushes on dental diseases. I. Quintessence Int..

[8] Tanna N, Kao E, Gladwin M, Ngan PW (2009). Effects of sealant and self-etching primer on enamel decalcification. Part I: an in-vitro study. Am. J. Orthod. Dentofac. Orthop..

[9] Roulet J-F, Zimmer S (2003). Prophylaxe und Präventivmedizin.

[10] Egelberg J, Claffey N. Role of mechanical dental plaque removal in prevention and therapy of caries and periodontal dieseases. Consensus Report of Group B. In: Lang, NP., Attsröm, R. & Löe, H., eds. Proceedings of the European Workshop on Mechanical Plaque Control. London.; 1998.

[11] Gale MS, Darvell BW (1999). Thermal cycling procedures for laboratory testing of dental restorations. J. Dent..

[12] Buskes JA, Christoffersen J, Arends J (1985). Lesion formation and lesion remineralization in enamel under constant composition conditions. A new technique with applications. Caries Res..

[13] Baumeister JP. Mikroradiografische Untersuchung zum EInfluss unterschiedlicher Lagerungsmedien und der POlitur auf die in-vitro-De- und Remineralisation von bovinen und humanem Schmelz [Dissertation]. Berlin: Charité - Universitiätsmedizin; 2011.

[14] Coordes SL. Vergleich verschiedener Präparate zum Prävention von Demineralisationen im Bracketumfeld [Dissertation]. Berlin: Charité - Unversitätsmedizin; 2013.

[15] Paschos E, Galosi T, Huth KC, Rudzki I, Wichelhaus A, Kunzelmann KH (2015). Do bonding agents protect the bracket-periphery?—Evaluation by consecutive μCT scans and fluorescence measurements. Clin. Oral Investig..

[16] Paschos E, Kleinschrodt T, Clementino-Luedemann T, Huth KC, Hickel R, Kunzelmann KH (2009). Effect of different bonding agents on prevention of enamel demineralization around orthodontic brackets. Am. J. Orthod. Dentofac. Orthop..

[17] Buren JL, Staley RN, Wefel J, Qian F (2008). Inhibition of enamel demineralization by an enamel sealant, pro seal: an in-vitro study. Am. J. Orthod. Dentofac. Orthop..

[18] Teruel Jde D, Alcolea A, Hernández A, Ruiz AJ (2015). Comparison of chemical composition of enamel and dentine in human, bovine, porcine and ovine teeth. Arch. Oral Biol..

[19] Kukleva MP, Shetkova DG, Beev VH (2002). Comparative age study of the risk of demineralization during orthodontic treatment with brackets. Folia Med. (Plovdiv)..

[20] Hu W, Featherstone JD (2005). Prevention of enamel demineralization: an in-vitro study using light-cured filled sealant. Am. J. Orthod. Dentofac. Orthop..

[21] Clark TJ. The efficacy of ProSeal TM, SeLECT Defense TM, OrthoCoat TM and Biscover LV TM resin sealants on the prevention of enamel demineralization abd white spot leasion formation. http//iruiowaedu/etd/479.: University of Iowa; 2010.

[22] Behnan SM, Arruda AO, González-Cabezas C, Sohn W, Peters MC. In-vitro evaluation of various treatments to prevent demineralization next to orthodontic brackets. Am J Orthod Dentofacial Orthop. 2010;138(6):712.e1–7; discussion -3. 10.1016/j.ajodo.2010.05.014 21130326

[23] Dyer D, Addy M, Newcombe RG (2000). Studies in vitro of abrasion by different manual toothbrush heads and a standard toothpaste. J. Clin. Periodontol..

[24] Slop D, Arends J (1987). Surface roughening of human enamel after toothbrushing. J. Biol. Buccale..

[25] Hotz P. [The abrasiveness of toothpastes]. Schweiz Monatsschr Zahnmed (1984). 1985;95(11):1066–7. 3866318

[26] Beals D, Ngo T, Feng Y, Cook D, Grau DG, Weber DA (2000). Development and laboratory evaluation of a new toothbrush with a novel brush head design. Am. J. Dent..

[27] Saxer UP, Barbakow J, Yankell SL (1998). New studies on estimated and actual toothbrushing times and dentifrice use. J. Clin. Dent..

[28] Cronin MJ, Dembling WZ, King DW, Goodman D, Cugini M, Warren PR (2002). A clinical study of plaque removal with an advanced rechargeable power toothbrush and a battery-operated device. Am. J. Dent..

[29] Buonocore MG (1981). Retrospections on bonding. Dent. Clin. N. Am..

[30] Boehm RF (1972). Thermal environment of teeth during open-mouth respiration. J. Dent. Res..

[31] Palmer DS, Barco MT, Billy EJ (1992). Temperature extremes produced orally by hot and cold liquids. J. Prosthet. Dent..

[32] Momoi Y, Iwase H, Nakano Y, Kohno A, Asanuma A, Yanagisawa K (1990). Gradual increases in marginal leakage of resin composite restorations with thermal stress. J. Dent. Res..

[33] Ingram GS, Silverstone LM (1981). A chemical and histological study of artificial caries in human dental enamel in vitro. Caries Res..

[34] Gray JA (1966). Kinetics of enamel dissolution during formation of incipient caries-like lesions. Arch. Oral Biol..

[35] Featherstone JD, Duncan JF, Cutress TW (1978). Crystallographic changes in human tooth enamel during in-vitro caries simulation. Arch. Oral Biol..

[36] Tschoppe P, Kielbassa AM, Toll R, Meyer-Lückel H (2009). Modification of the mineralizing capacity of a saliva substitute (saliva natura) on enamel in vitro. Laryngorhinootologie..

[37] Arends J, ten Bosch JJ (1992). Demineralization and remineralization evaluation techniques. J. Dent. Res..

[38] Paschos E, Rosenbeck KA, Huth KC, Rudzki I, Wichelhaus A, Kunzelmann KH (2015). Evaluation of the effect of bracket-periphery treatment on prevention of enamel demineralization by consecutive μCT scans. Clin. Oral Investig..

[39] Silin AV, Satygo EA, Sadal'skiĭ IuS (2014). Efficacy of the caries preventive agents in children during mixed dentition period. Stomatologiia (Mosk)..

[40] Shinaishin SF, Ghobashy SA, El-Bialy TH (2011). Efficacy of light-activated sealant on enamel demineralization in orthodontic patients: an atomic force microscope evaluation. Open Dent. J..

[41] Cain K, Hicks J, English J, Flaitz C, Powers JM, Rives T (2006). In vitro enamel caries formation and orthodontic bonding agents. Am. J. Dent..

[42] Heinig N, Hartmann A (2008). Efficacy of a sealant : study on the efficacy of a sealant (Light Bond) in preventing decalcification during multibracket therapy. J. Orofac. Orthop..

[43] Frazier MC, Southard TE, Doster PM (1996). Prevention of enamel demineralization during orthodontic treatment: an in vitro study using pit and fissure sealants. Am. J. Orthod. Dentofac. Orthop..

[44] Wilson RM, Donly KJ (2001). Demineralization around orthodontic brackets bonded with resin-modified glass ionomer cement and fluoride-releasing resin composite. Pediatr. Dent..

[45] Seppä L, Tuutti H, Luoma H (1982). Three-year report on caries prevention of using fluoride varnishes for caries risk children in a community with fluoridated water. Scand. J. Dent. Res..

[46] Shafi I (2008). Fluoride varnish reduces white spot lesions during orthodontic treatment. Evid. Based Dent..

[47] Stecksén-Blicks C, Renfors G, Oscarson ND, Bergstrand F, Twetman S (2007). Caries-preventive effectiveness of a fluoride varnish: a randomized controlled trial in adolescents with fixed orthodontic appliances. Caries Res..

[48] He WD, Liu YZ, Xu YY, Chen D (2010). Study on application of CPP-ACP on tooth mineralization during orthodontic treatment with fixed appliance. Shanghai Kou Qiang Yi Xue..

[49] de Bruyn H, Buskes JA, Arends J (1986). The inhibition of demineralization of human enamel after fluoride varnish application as a function of the fluoride content. An in vitro study under constant composition demineralizing conditions. J. Biol. Buccale..

[50] Bechtold TE, Sobiegalla A, Markovic M, Berneburg M, Göz GR (2013). In vivo effectiveness of enamel sealants around orthodontic brackets. J. Orofac. Orthop..

